# *Interleukin-23R* rs7517847 T/G Polymorphism Contributes to the Risk of Crohn's Disease in Caucasians: A Meta-Analysis

**DOI:** 10.1155/2015/279849

**Published:** 2015-05-18

**Authors:** Li Zhang, Yunjie Lu, Yuzheng Ge, Yun Shi, Xing Wu, Qinghua Xu, Xiaoping Li, Ling Lu, Feng Zhang, Guozhong Yao

**Affiliations:** ^1^Liyang People's Hospital, Liyang 213300, China; ^2^Translational Medicine Research Center of Jiangning Hospital and Liver Transplantation Center of First Affiliated Hospital, Nanjing Medical University, Nanjing 210029, China; ^3^Department of Urology, Nanjing First Hospital, Nanjing Medical University, Nanjing 210029, China

## Abstract

The association between *Interleukin-23R* gene polymorphism and Crohn's disease (CD) in Caucasians is still controversial. Thus, a meta-analysis was performed to evaluate the correlation between this gene variant and CD risk. We retrieved the available data from EMBASE and PUBMED until May 1, 2014, and evaluated the effect of rs7517847 in Caucasians. The significant associations were confirmed between rs7517847 and CD risk in dominant models (TT/TG versus GG: OR = 1.652, 95% CI 1.277, 2.137), allelic model (T allele versus G allele: OR = 1.327, 95% CI 1.198, 1.469), homozygote comparison (TT versus GG: OR = 1.890, 95% CI 1.465, 2.437), heterozygote comparison (TG versus GG: OR = 1.509, 95% CI 1.161, 1.960), and recessive model (TT versus TG/GG: OR = 1.409, 95% CI 1.279, 1.552). In conclusion, this meta-analysis demonstrates that rs7517847 is associated with the risk of CD in Caucasians. These findings show that IL-23R genes confer susceptibility to CD in the Caucasians.

## 1. Introduction

Crohn's disease (CD) is a form of inflammatory bowel disease (IBD) that primarily affects the Caucasian population [1, 2]. It is a heritable disease which is influenced by many genetic risk factors [3]. Therefore, identification of gene risk factors of CD is beneficial for the clinical treatment of patients.

Interleukin 23 (IL-23) plays an important role in the inflammatory response against infection as a regulator of immune cells [4]. IL-23R which interacts with IL-23 is a protein consisting of an IL-12*β*1 and an IL-23R chain [5]. Recently, the mechanisms of IL-23R variants have been investigated in different autoimmune diseases [6–9]. Studies also have shown that rs7517847, the single nucleotide polymorphisms (SNPs) of the* IL23R* gene, are associated with CD occurring rate [10, 11]. However, the association between IL-23R polymorphisms and CD susceptibility are inconclusive and controversial due to small sample size in each of the published studies.

To better understand the association of IL-23R polymorphisms and CD susceptibility in Caucasians, we conducted a meta-analysis of all eligible studies and hope to yield more accurate and robust estimates.

## 2. Materials and Methods

### 2.1. Search Strategy

We searched for relevant studies in the following databases: EMBASE and PUBMED. Available studies for IL-23R polymorphism and CD were collected by different combinations of various key words: Interleukin-23 receptor, IL-23R; polymorphism, variant, or mutation; Crohn's disease, CD. Languages restriction was not imposed in this research and only published studies with full text were included in this meta-analysis.

### 2.2. Inclusion and Exclusion Criteria of Trials

In the meta-analysis, the following inclusive selection criteria were set: (a) study design evaluating the association between IL-23R polymorphism and CD risk; (b) case control design; (c) Caucasians design. The following exclusive selection criteria were set: (a) no control cases; (b) duplication of the previous publication; (c) no available genotype frequency; for studies with overlapped or repeated data (d) no Caucasians.

### 2.3. Data Extraction

Eligible studies were extracted by 2 reviewers (Li Zhang and Yunjie Lu) independently according to the predesigned data collection form. The following information was extracted: first author's name, publication year, country, ethnicity, immune suppressive protocol, number of cases and controls, and genotype distribution in both groups. Disagreement was resolved by discussion with a third reviewer (Guozhong Yao).

### 2.4. Statistical Analysis

For each trial, odds ratio (OR) with the 95% confidence interval (95% CI) of the survival rate was derived and calculated. Increased or decreased risk of CD was indicated by 95% CI without 1 for OR. The pooled ORs were estimated for allelic model (T allele versus G allele), homozygote comparison (TT versus GG) and heterozygote comparison (TG versus GG), dominant models (TT/TG versus GG), and recessive model (TT versus TG+GG). *Z* test was performed to assess the significance of the pooled OR. Between-study heterogeneity was assessed by the Cochran's *Q* statistic and *I* ^2^ tests [12]. The random effects model was conducted if the *Q* test exhibited a *P* < 0.05 or the *I* ^2^ test showed >50%. Otherwise, the fixed effects model would be conducted. For publication bias, the Begg's funnel plot and Egger's linear regression test were conducted, and *P* < 0.05 was considered significant.

A fixed-effect model (based on Mantel-Haenszel method) was utilized to pool the data from different studies if the between-study heterogeneity was absent, or a random-effect model (based on DerSimonian and Laird method) was applied.

The statistical analysis was performed by STATA 10.0 (Stata Corp LP, College Station, TX, USA). All *P* values are two-side.

## 3. Results

### 3.1. Selection of the Included Studies and Characteristics

**Figure 1:**
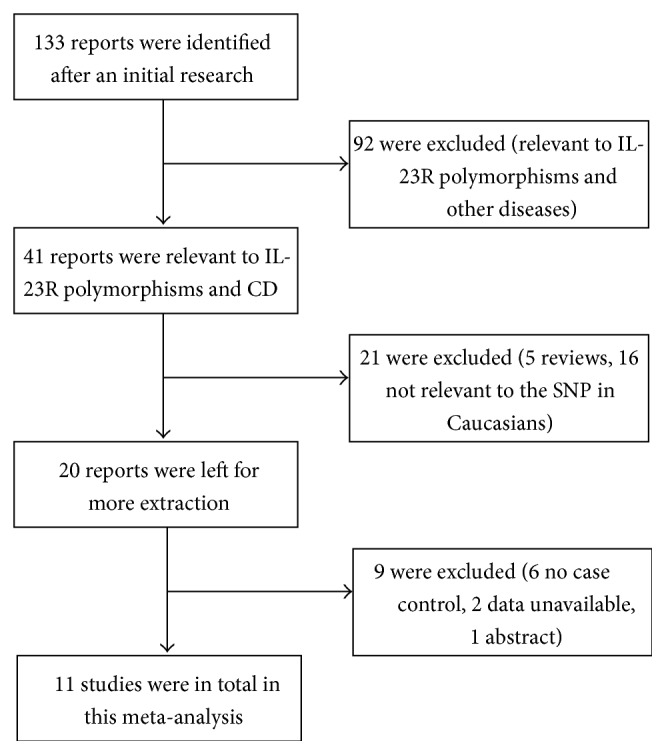
Flow diagram of the study selection process and specific reasons for exclusion.

The flow diagram of studies selection and exclusion reasons were represented in Figure 1. A total of 133 studies were identified by our first research; a number of 41 were preliminarily yielded out after excluding inappropriate studies and screening abstract-screening, full-text assessment. In these 41 studies, 30 were excluded, 11 articles containing rs7517847 in Caucasians were recruited for detailed analysis (Table 1), and these data built Table 1 [13–23]. Each of them was independent. Thus, a total of 3279 CD cases and 4136 healthy controls were included in our meta-analysis. All of them were Caucasian and the diagnosis of CD was based on clinical manifestations and laboratory examinations and further biopsy.

### 3.2. Evaluation of the Association

**Figure 2:**
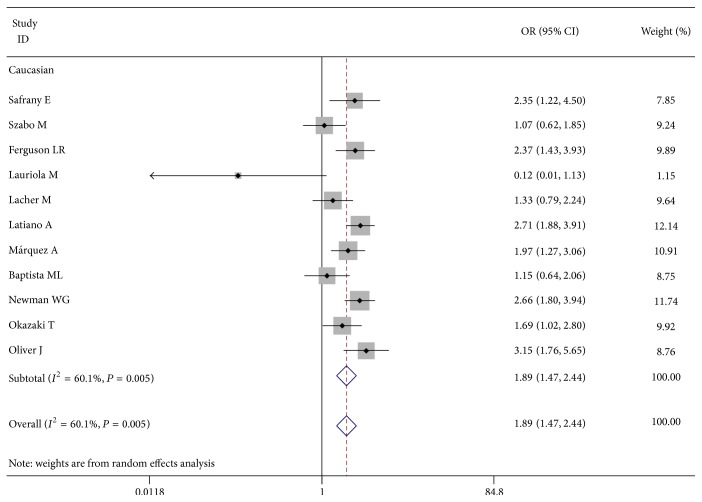
Forest plot for risk of CD associated with rs7517847 in Caucasian (TT versus GG. For each study, the estimate of OR and its 95% CI is plotted with a box and a horizontal line. Filled diamond pooled OR and its 95% CI).

The OR from all models indicated a significant association between rs7517847 and CD. After pooling all the eligible studies in Table 2, we found that the risk of CD was significantly associated with rs7517847 in dominant models (TT/TG versus GG: OR = 1.652, 95% CI 1.277, 2.137), allelic model (T allele versus G allele: OR = 1.327, 95% CI 1.198, 1.469), homozygote comparison (TT versus GG: OR = 1.890, 95% CI 1.465, 2.437, Figure 2), heterozygote comparison (TG versus GG: OR = 1.509, 95% CI 1.161, 1.960), and recessive model (TT versus TG/GG: OR = 1.409, 95% CI 1.279, 1.552). These data demonstrate that rs7517847 increases the risk of CD among Caucasians with hospital-based studies.

### 3.3. Publication Bias

**Figure 3:**
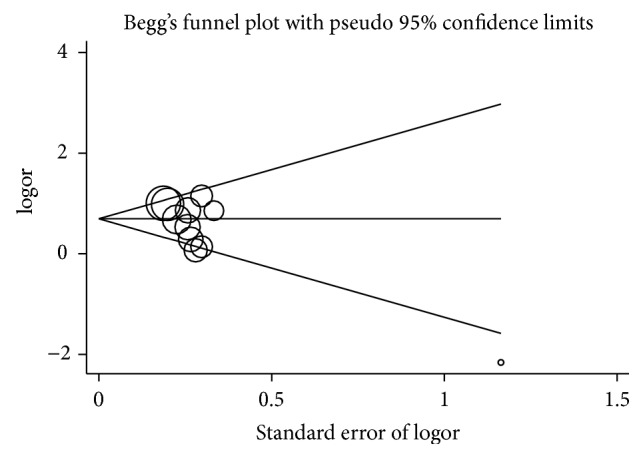
Begg's funnel plot for publication bias test (TT versus GG for rs7517847).

Begg's funnel plot and Egger's test were both performed to assess the publication bias of this meta-analysis. The shape of the funnel plots for homozygote comparison models seemed symmetrical (Figure 3). Then, the Egger's test was used to provide statistical evidence of funnel plot symmetry. The results still did not suggest any evidence of publication bias. Thus, publication bias was not evident in present meta-analyses.

## 4. Discussion

CD is associated with JAK2 signaling pathway which is activated by IL-23 and IL-23R receptor [24]. Previous studies suggested that the interruption of IL-23R SNPs might lead to the dysregulation of intestinal inflammation [25]. IL-23R gene variants also play an essential role in the development of many autoimmune diseases such as ankylosing spondylitis (AS), inflammatory bowel disease (IBD), and systemic lupus erythematosus (SLE) [7, 26, 27]. Therefore, researchers are focusing on observing the relationship between IL-23R gene polymorphisms and the risk of CD. However, the results are conflicting and controversial due to the different races and insufficient sample size. After pooling data for 11 studies in this meta-analysis, our results firstly demonstrate that T allele of rs7517847 was highly susceptible to CD in Caucasians.

One previous study showed that rs7517847 is a protective factor in rheumatoid arthritis (RA) in European population. Interestingly, RA is a systemic autoinflammatory disease which is associated with PTPN22/C1858T, while the organ-specific autoimmune disease CD is not [28, 29]. Thus, the mechanism of this genetic variant may not play a common role in different autoimmune diseases. More researches are required to observe the exact mechanisms of IL-23R gene polymorphism.

We should also mention the limitations of this meta-analysis. Primarily, all the studies were limited to the Caucasian. The allelic frequencies may be different in other ethnic groups. Secondly, publication bias might occur even if there is no significance in statistical test due to extracting published studies. Ultimately, owing to methodological limitations, this meta-analysis is retrospective. Two independent authors performed the process of study selection and data extraction and a third author resolved the discrepancy to minimize the bias.

In conclusion, our meta-analysis suggests that IL-23R rs7517847 confers susceptibility to CD in the Caucasians. Furthermore, more studies with larger scale are required to confirm these associations.

## Figures and Tables

**Table 1:** Characteristics of eligible studies included in the meta-analysis.

Author	Year	Country	Ethnicity	Genotyping methods	Case			Control			HWE
					TT	TG	GG	TT	TG	GG	
Safrany et al. [13]	2013	Hungary	Caucasian	PCR–RFLP	72	110	17	74	138	41	0.081
Szabo et al. [14]	2013	Hungary	Caucasian	PCR–RFLP	150	182	64	57	99	26	0.104
Ferguson et al. [15]	2010	New Zealand	Caucasian	MassARRAY	108	172	29	113	183	72	0.892
Lauriola et al. [16]	2011	Italy	Caucasian	PCR	9	4	6	13	6	1	0.78
Lacher et al. [17]	2010	Germany	Caucasian	RT-PCR	81	101	39	78	125	50	0.995
Latiano et al. [18]	2008	Italy	Caucasian	PCR-RFLP	366	305	52	280	328	108	0.459
Márquez et al. [19]	2008	Spain	Caucasian	RT-PCR	145	161	36	192	260	94	0.71
Baptista et al. [20]	2008	Brazil	Caucasian	RT-PCR	59	95	28	79	120	43	0.825
Newman et al. [21]	2009	England	Caucasian	MassARRAY	195	204	40	300	436	164	0.799
Okazaki et al. [22]	2008	Canada	Caucasian	RT-PCR	84	91	36	91	157	66	0.91
Oliver et al. [23]	2007	Spain	Caucasian	Taqman PCR	101	119	18	121	153	68	0.124

**Table 2:** Stratified analysis of rs7517847 polymorphism and CD risk in eligible studies.

Author	Year	T versus G allele	TT versus GG OR (95% CI)	TG versus GG OR (95% CI)	TT + TG versus GG (95% CI)	TT versus TG + GG (95% CI)
**Total**		**1.327 (1.198, 1.469)**	**1.890 (1.465, 2.437)**	**1.509 (1.161, 1.960) **	**1.652 (1.277, 2.137)**	**1.409 (1.279, 1.552)**
Safrany et al. [13]	2013	1.357 (1.036, 1.777)	2.347 (1.223, 4.503)	1.922 (1.036, 3.568)	2.070 (1.137, 3.769)	1.371 (0.923, 2.038)
Szabo et al. [14]	2013	1.102 (0.856, 1.419)	1.069 (0.618, 1.850)	0.747 (0.445, 1.253)	0.865 (0.528, 1.417)	1.337 (0.921, 1.942)
Ferguson et al. [15]	2010	1.349 (1.084, 1.678)	2.373 (1.432, 3.933)	2.334 (1.446, 3.766)	2.349 (1.481, 3.724)	1.213 (0.879, 1.673)
Lauriola et al. [16]	2011	0.344 (0.126, 0.941)	0.115 (0.012, 1.129)	0.111 (0.009, 1.309)	0.114 (0.012, 1.062)	0.485 (0.134, 1.754)
Lacher et al. [17]	2010	1.176 (0.908, 1.523)	1.331 (0.790, 2.243)	1.036 (0.632, 1.698)	1.149 (0.723, 1.828)	1.298 (0.886, 1.902)
Latiano et al. [18]	2008	1.553 (1.328, 1.816)	2.715 (1.884, 3.913)	1.931 (1.340, 2.784)	2.292 (1.618, 3.248)	1.596 (1.295, 1.968)
Márquez et al. [19]	2008	1.347 (1.104, 1.643)	1.972 (1.269, 3.063)	1.617 (1.050, 2.489)	1.768 (1.172, 2.665)	1.357 (1.029, 1.791)
Baptista et al. [20]	2008	1.045 (0.793, 1.377)	1.147 (0.640, 2.055)	1.216 (0.704, 2.100)	1.188 (0.706, 2.000)	0.990 (0.656, 1.492)
Newman et al. [21]	2009	1.542 (1.302, 1.827)	2.665 (1.805, 3.935)	1.918 (1.307, 2.815)	2.223 (1.541, 3.207)	1.598 (1.265, 2.019)
Okazaki et al. [22]	2008	1.355 (1.054, 1.741)	1.692 (1.024, 2.798)	1.918 (1.307, 2.815)	1.294 (0.825, 2.029)	1.621 (1.122, 2.342)
Oliver et al. [23]	2007	1.515 (1.187, 1.935)	3.153 (1.761, 5.648)	2.938 (1.658, 5.207)	3.033 (1.752, 5.252)	1.347 (0.959, 1.891)
